# Effects of genetic strain, stocking density, and age on broiler behavior

**DOI:** 10.1016/j.psj.2024.104723

**Published:** 2024-12-24

**Authors:** Rosemary H. Whittle, Darrin M. Karcher, Marisa A. Erasmus, Shawna L. Weimer

**Affiliations:** a Department of Poultry Science, University of Arkansas, Fayetteville, Arkansas, USA 72701; b Department of Animal Sciences, Purdue University, West Lafayette, Indiana, USA 47907

**Keywords:** Broiler, Slow growing, Stocking density, Behavior, Welfare

## Abstract

Fast growth rate and stocking density are global animal welfare concerns for broiler chickens. The objective of this study was to evaluate the effect of genetic strain and stocking density on the behavior of broilers. In a 2 × 2 randomized complete block design, conventional (CONV) and slow-growing (SG) broilers were stocked at either 29 kg/m^2^ (LO, *n* = 31 birds/pen) or 37 kg/m^2^ (HI, *n* = 40 birds/pen) in 16 pens (*n* = 4 pens/treatment). On days 25 and 39 (CONV and SG), and 60 (SG only), behavior was observed from video recorded in the morning and afternoon each day. The percentage (%) of all birds in each pen was categorized as either walking, standing, sitting, lateral sitting, eating, drinking, or preening. Two data sets were generated to compare the effect of age (25, 39d) and market body weight (39d CONV, 60d SG). Linear mixed effects models were fitted in R to analyze data. Sitting behavior differed between broiler strains and ages. At 39d, more CONV sat compared to 25d (58.3 % vs 54.5 %, *p* < 0.0001) and compared to SG at market weight (58.3 % vs 43.9 %, *p* < 0.0001). CONV broilers sat in a lateral posture more than SG at both ages (5.4 % vs 1.4 %, *p* < 0.0001) and at market weight (7.4 % vs 0.4 %, *p* < 0.0001). Standing and walking behaviors were observed more in SG broilers. SG broilers walked more than CONV at 39d and at market weight (2.4 % vs 1.6, *p* ≤ 0.01). Further, SG broilers stood more than CONV at both ages (11.4 % vs. 7.2 %, *p* = 0.0004) and market weight (14.9 % vs. 7.1 %, *p* < 0.0001). While preening behavior did not differ at 25d, more SG broilers preened than CONV at 39d (5.6 % vs 3.9 %, *p* < 0.0001) and market weight (5.5 % vs 3.3 %, *p* < 0.0001). LO-stocked broilers preened more at both ages than at HI (5.6 % vs 5.1 %, *p* = 0.041). These results suggest that conventional broilers exhibit more sitting behaviors, slow-growing broilers exhibit more active behaviors, and chronological and physiological age differences should be considered when making comparisons.

## Introduction

Slow-growing broilers have recently gained traction in mainstream broiler production in some European countries due to their improved leg health and welfare compared to conventional broilers (Rayner et al., 2020; Abeyesinghe et al., 2021; Baxter et al., 2021). Slow-growing broilers have been shown to have a better gait, lower prevalence of hock burn and footpad dermatitis (Rayner et al., 2020; Baxter et al., 2021), and perform more behaviors associated with positive welfare such as foraging (Dixon, 2020; Rayner et al., 2020; van der Eijk et al., 2022), dustbathing (Dixon, 2020; Rayner et al., 2020; Ghayas et al., 2021) and play (Dixon, 2020; Rayner et al., 2020). However, in the US, slow-growing broilers represent a small market share. Slow-growing broilers have lower breast yield, reduced feed efficiency, and take longer to achieve market weight compared to conventional broilers (Weimer et al., 2020, 2022; Rayner et al., 2020; Baxter et al., 2021). These factors result in higher costs to producers and consumers (Lusk et al., 2019). While many companies are committing to providing chicken products from broilers raised with higher welfare standards, this shift does not yet include transitioning to slower-growing breeds. Nonetheless, some animal welfare certification programs specify the genetic strain of broilers required. For example, the Global Animal Partnership (GAP), an animal welfare certification organization, lists approved slow- and moderate-growing broiler strains required for certification (GAP, 2024). However, research directly comparing conventional and slow-growing broiler behavior is limited, particularly comparing strains at the same body weight.

Previous research has reported that conventional and slow-growing broilers behave differently. Slow-growing broilers tend to be more active, showing increased energy-intensive behaviors such as walking, foraging, and dustbathing, whereas conventional broilers tend to be more inactive, spending more time sitting (Bokkers and Koene, 2003; Wallenbeck et al., 2016; Dixon, 2020; Dawson et al., 2021; Zhou et al., 2024; Guinebretière et al., 2024). Larger body size coupled with a high resting metabolic rate constrains the locomotor activity of conventional broilers (Tickle and Codd, 2019). Research into the behavior of slow-growing broiler chickens is ongoing; there is relatively little published research exploring the behavior of slow-growing broilers and directly comparing them to conventional broilers at the same age or body weight in the same housing conditions.

In the U.S., industry-wide guidelines on commercial broiler stocking density include the National Chicken Council's recommendation that broiler flocks be stocked at densities ranging between 32 and 44 kg/m^2^, depending on market weight (NCC, 2022). Some broiler welfare programs require broilers to be housed at lower stocking densities than conventional practices. For example, in section 7 CFR 205.241(c)(6) the National Organic Program (NOP) stipulates that broilers must be stocked at a maximum density of 24.4 kg/m^2^ indoors in addition to being provided with outdoor access (NOP, 2023). In contrast, European countries have a legislative approach, with the European Union Council Directive 2007/43/EC stipulating that broiler stocking density cannot exceed 33 kg/m^2^ (Council of the European Union, 2007). An exemption to allow an increase to 39 kg/m^2^ can be given when additional documented details for each house are kept, and the house achieves certain climatic parameters. A further increase to 42 kg/m^2^ is allowed where, in addition to meeting the aforementioned conditions, monitoring by the authorities confirms records of low mortality rates and good management practices (Council of the European Union, 2007).

One challenge when studying the effects of stocking density and broiler behavior is that environmental management contributes more to broiler welfare than stocking density *per se* (Dawkins et al., 2004). However, some studies demonstrated that housing broilers at lower stocking densities improved key indicators of animal welfare, including decreasing the prevalence of hock burn and pododermatitis and improving leg health and gait (Dixon, 2020; Weimer et al., 2020; Rayner et al., 2020; Baxter et al., 2021; van der Eijk et al., 2022). In contrast, relatively higher stocking densities have been shown to limit the behavioral repertoire of broiler chickens, with broilers that are kept at low densities showing increased foraging and play behavior (Rayner et al., 2020; van der Eijk et al., 2022; Evans et al., 2023). However, results are inconsistent, with some studies indicating broiler chickens behave differently at lower stocking densities (Rayner et al., 2020; van der Eijk et al., 2022; Evans et al., 2023), and others finding little to no effect (Tahamtani et al., 2018; Zhou et al., 2024; Guinebretière et al., 2024). Further, the effects of stocking density on positive behaviors are largely unknown.

Previous work has identified that, while slow-growing broiler behavior clearly differs from the behavior of conventional broilers, these differences are not always consistent and can vary with stocking density (van der Eijk et al., 2022), indicating that slower-growing strains of broilers respond differently to stocking density. Given that genetics and environment influence behavior, the objective of this study was to evaluate the effect of stocking density on the behavior of conventional and slow-growing broilers raised indoors. We hypothesized more active behaviors would be observed at lower stocking densities and that slow-growing broilers would display more active behaviors compared to conventional broilers, whilst conventional broilers would display more inactive behaviors.

## Materials and methods

### Animals and housing

The Purdue Institutional Animal Care and Use Committee approved the experimental design and procedures. Male broilers (*n* = 568) of two genetic strains were raised to a 2.8 kg market weight, one conventional (CONV, Ross 708, *n* = 284) with a 42d market weight and one slow-growing (SG, Hubbard Redbro, *n* = 284) with a 63d market weight. Broilers were housed in 16 pens (1.5 m x 2.4 m; 4 per strain/ stocking density) in a 2 × 2 factorial complete randomized block design. At day-of-hatch, broilers of each strain were exclusively placed into pens at two stocking densities, 29 kg/m^2^ (LO, *n* = 31 birds/pen) and 37 kg/m^2^ (HI, *n* = 40 birds/pen). Broilers were fed standard commercial rations in three phases: starter (3187 kcal/kg ME; 230 g/kg protein), grower (3121 kcal/kg ME; 221 g/kg protein), and finisher (3283 kcal/kg ME; 201 g/kg protein). The temperature curve was set to 33°C at one day of age and then decreased incrementally to 21°C by day 27. The photoperiod was initially 23 h light: 1 h dark and dark hours were added gradually until day 14 when 18 h light: 6 h dark was achieved.

### Sampling

Sixteen overhead CCTV cameras (Geovision, Irvine, CA) were mounted above each pen, and recordings were taken on days 25, 39, and 60 (SG only) for 30 min in the morning (0630) and 30 min in the afternoon (1400). Video recordings were analyzed using instantaneous scan sampling at 30-second intervals (124 scans/pen/age). These sampling intervals are typical of broiler behavior research (Bokkers and Koene, 2003; Dixon, 2020; de Jong et al., 2021). At each scan interval, the numbers of broilers performing each behavior - walking, standing, sitting, lateral sitting, eating, drinking, preening, foraging, and dustbathing - were recorded following the ethogram in Table 1. Behavioral observations were recorded by three observers and inter-observer reliability with a test set of videos before observations was 83 %.

**Table 1:** Ethogram of behaviors.

**Behavior**	**Description**
Walking	relatively low speed of displacement of the bird on the ground in which propulsive force is derived from the action of the legs
Standing	bird maintains an upright position on extended legs
Sitting	hocks and/or breast resting on the ground, head may or may not be on the ground
Lateral sitting	sitting with the left or right leg extended away from the body, the bird may lean to the opposite side of the extended leg
Eating	head above the feeder looking down at feed, may or may not be actively pecking at feed
Drinking	pecking down into the bell drinker; birds may tip their head back to swallow
Preening	the act of pecking, nibbling, stroking, or combing plumage with the beak
Foraging	scratching with feet and/or pecking at the ground with the beak while standing
Dustbathing	the act of building a dirt mound using feet, wings, and beak and then lying on the ground and tossing substrate on its back and wings, typically birds will distribute the substrate into feathers with vertical wing shakes

### Statistical analysis

Data were analyzed as the percentage (%) of broilers per pen engaged in each behavior for each scan. The data were split into two data sets, one comparing CONV and SG at two ages (25d and 39d) and the other comparing 39d CONV and 60d SG at the same market body weight. Foraging and dustbathing behaviors were not statistically analyzed due to low prevalence. Behaviors were analyzed individually in Rv4.4.0 and R Studio using “lme4” package to create linear mixed effects models where estimated means were generated using “emmeans” package. Models were assessed for residual normality using qqplots and Shapiro-wilks tests for normality.

For the age comparisons genetic strain (CONV, SG), stocking density (LO, HI), age (25d, 39d), and their interactions were used as fixed effects with time of day (AM, PM) as a covariate. Walking, standing, sitting, lateral sitting, and drinking were not normally distributed and were square-root transformed to improve model fit, and estimated means were back-transformed. Pens were grouped into blocks based on their location and pen nested in block was used as a random effect for walking, lateral sitting, and preening. Pen was used as a random effect for standing, sitting, eating, and drinking, whereas block was removed as a random effect due to not significantly accounting for variation of the data.

For the market body weight comparison, genetic strain (CONV, SG), stocking density (LO, HI), and their interaction were used as fixed effects with time of day as a covariate. Walking, sitting, eating, and drinking were square-root transformed to improve model fit. Pen nested in block was used as a random effect for walking, standing, sitting, lateral sitting, eating, drinking, and preening.

## Results

**Fig. 1:**
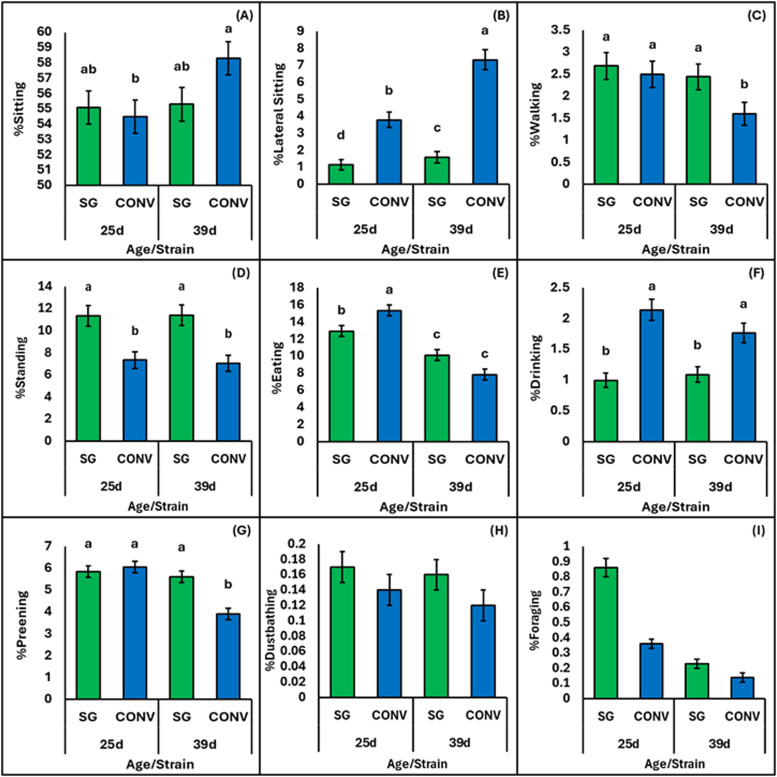
Age comparison for the percentage of conventional (CONV) and slow-growing (SG) broilers at two ages (25 and 39 days) observed (A) sitting, (B) lateral sitting, (C) walking, (D) standing, (E) eating, (F) drinking, (G) preening, (H) dustbathing, and (I) foraging. A-G shows estimated means and significant pairwise comparisons (*p* < 0.05) between strain and age are shown with differing superscript letters. H-I were too infrequent to be analyzed statistically, therefore, raw means and standard error are provided.

**Fig. 2:**
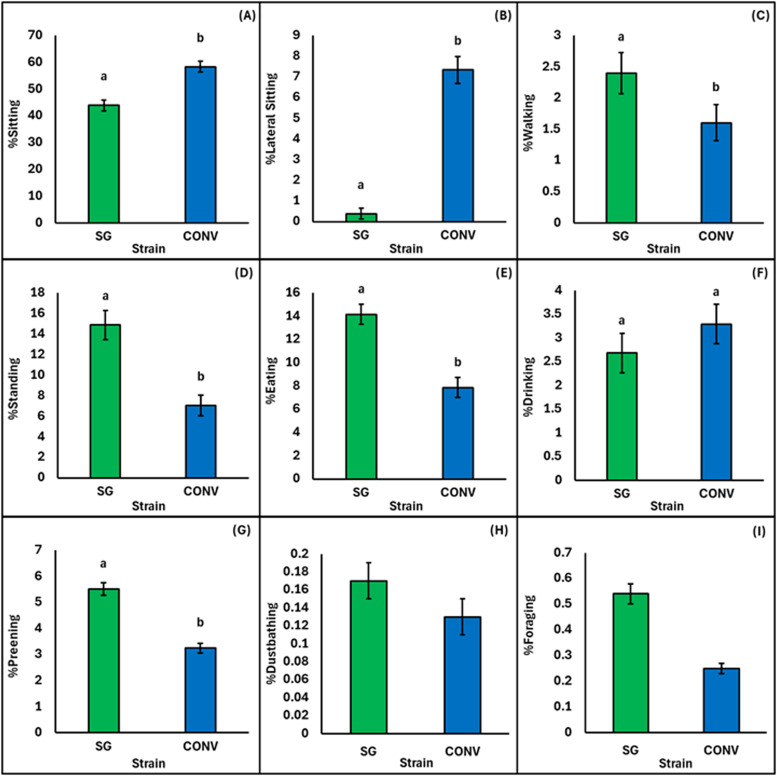
Market body weight comparison for the percentage of conventional (CONV) and slow-growing (SG) broilers, (A) sitting, (B) lateral sitting, (C) walking, (D) standing, (E) eating, (F) drinking, (G) preening, (H) dustbathing, and (I) foraging. A-G shows estimated means and significant pairwise comparisons (*p* < 0.05) between strains are shown with differing superscript letters. H-I were too infrequent to be analyzed statistically, therefore, raw means and standard error are provided.

The results presented in Fig. 1A–I report the mean percentage of CONV and SG broilers observed sitting, lateral sitting, walking, standing, eating, drinking, preening, dustbathing, and foraging on 25d and 39d. The results presented in Fig. 2A–I report the mean percentage of 39d CONV and 60d SG broilers, matched for body weight, observed sitting, lateral sitting, walking, standing, eating, drinking, preening, dustbathing, and foraging. Dustbathing and foraging were observed infrequently and, therefore, were not statistically analyzed, and the raw means are presented. Table 2 reports the estimated mean percentage, chi-square test statistic, and p-value for strain, stocking density, age, and their interactions of age-matched (25d and 39d) CONV and SG broilers observed walking, standing, sitting, lateral sitting, eating, drinking, and preening. Table 3 reports the corresponding results for these behaviors based on body weight-matched comparisons of 39d CONV and 60d SG broilers; there were no interactions found between strain and stocking density (*p* > 0.05), and results are only reported for the main effect of stocking density.

**Table 2:** Estimated means (±SE) for the percentage of conventional (CONV) and slow growing (SG) broilers housed at two stocking densities (LO, HI)performing different behaviors at 25 and 39 days. Significant results are bolded and significant pairwise comparisons are depicted using differing superscript letters. Dustbathing and foraging were observed too infrequently for statistical analysis; therefore, raw means and standard errors are reported.

Behavior									*χ^2^*	*p*
**Sitting**										
Strain	CONV	SG								
	56.4 ± 1.07	55.2 ± 1.07							0.60	0.44
Stocking Density	LO	HI								
	55.6 ± 1.07	55.9 ± 1.07							0.05	0.83
Age	25d	39d								
	54.8 ± 0.77^b^	56.8 ± 0.77^a^							**33.88**	**<0.0001**
Strain Stocking Density	CONVLO	CONVHI	SGLO	SGHI						
	55.5 ± 1.51	57.3 ± 1.51	55.8 ± 1.51	54.6 ± 1.51					0.96	0.33
Stocking Density	LO	LO	HI	HI						
Age	25d	39d	25d	39d						
	54.6 ± 1.09	56.6 ± 1.09	54.9 ± 1.09	57.0 ± 1.09					0.003	0.95
Strain Stocking Density	CONVLO	CONVLO	CONVHI	CONVHI	SGLO	SGLO	SGHI	SGHI		
Age	25d	39d	25d	39d	25d	39d	25d	39d		
	53.6 ± 1.55	57.3 ± 1.55	55.2 ± 1.55	59.3 ± 1.55	55.6 ± 1.55	56.0 ± 1.55	54.6 ± 1.55	54.7 ± 1.55	0.27	0.60
**Lateral Sitting**									*χ^2^*	*p*
Strain	CONV	SG								
	5.4 ± 0.52^a^	1.4 ± 0.31^b^							**47.83**	**<0.0001**
Stocking Density	LO	HI								
	3.4 ± 0.43	2.9 ± 0.40							0.61	0.43
Age	25d	39d								
	2.4 ± 0.27^b^	4.1 ± 0.33^a^							**305.09**	**<0.0001**
Strain Stocking Density	CONVLO	CONVHI	SGLO	SGHI						
	5.6 ± 0.74	5.5 ± 0.72	1.6 ± 0.47	1.1 ± 0.42					0.16	0.69
Stocking Density	LO	LO	HI	HI						
Age	25d	39d	25d	39d						
	2.5 ± 0.39^bc^	4.4 ± 0.48^c^	2.2 ± 0.37^a^	3.7 ± 0.45^ab^					3.38	0.07
Strain Stocking Density	CONVLO	CONVLO	CONVHI	CONVHI	SGLO	SGLO	SGHI	SGHI		
Age	25d	39d	25d	39d	25d	39d	25d	39d		
	3.8 ± 0.64	7.7 ± 0.86	3.8 ± 0.64	7.0 ± 0.83	1.4 ± 0.45	1.9 ± 0.50	1.0 ± 0.41	1.3 ± 0.44	0.25	0.62
**Walking**									*χ^2^*	*p*
Strain	CONV	SG								
	2.04 ± 0.27^b^	2.57 ± 0.29^a^							**64.96**	**0.026**
Stocking Density	LO	HI								
	2.24 ± 0.13	1.75 ± 0.30							0.01	0.91
Age	25d	39d								
	2.60 ± 0.27	2.01 ± 0.25							**38.88**	**<0.0001**
Strain Stocking Density	CONVLO	CONVHI	SGLO	SGHI						
	2.12 ± 0.32	1.95 ± 0.31	2.44 ± 0.34	2.69 ± 0.35					0.77	0.38
Stocking Density	LO	LO	HI	HI						
Age	25d	39d	25d	39d						
	2.44 ± 0.30^ab^	2.13 ± 0.28^ab^	2.76 ± 0.28^a^	1.89 ± 0.27^b^					**8.36**	**0.0038**
Strain Stocking Density	CONVLO	CONVLO	CONVHI	CONVHI	SGLO	SGLO	SGHI	SGHI		
Age	25d	39d	25d	39d	25d	39d	25d	39d		
	2.28 ± 0.34^a^	1.97 ± 0.32^ab^	2.73 ± 0.36^a^	1.26 ± 0.56^b^	2.29 ± 0.34^a^	2.29 ± 0.34^a^	2.78 ± 0.37^a^	2.60 ± 0.36^a^	**12.72**	**0.0004**
**Standing**									*χ^2^*	*p*
Strain	CONV	SG								
	7.19 ± 0.74^b^	11.37 ± 0.91^a^							**12.69**	**0.0004**
Stocking Density	LO	HI								
	8.80 ± 0.81	9.56 ± 0.844							0.42	0.52
Age	25d	39d								
	9.23 ± 0.60	9.11 ± 0.59							0.31	0.58
Strain Stocking Density	CONVLO	CONVHI	SGLO	SGHI						
	7.28 ± 1.06	7.10 ± 1.05	10.44 ± 1.24	12.33 ± 1.34					0.67	0.41
Stocking Density	LO	LO	HI	HI						
Age	25d	39d	25d	39d						
	9.18 ± 0.84^a^	8.42 ± 0.81^a^	9.29 ± 0.85^a^	9.82 ± 0.87^a^					**8.51**	**0.0035**
Strain Stocking Density	CONVLO	CONVLO	CONVHI	CONVHI	SGLO	SGLO	SGHI	SGHI		
Age	25d	39d	25d	39d	25d	39d	25d	39d		
	7.56 ± 1.09	7.01 ± 1.06	7.12 ± 1.07	7.09 ± 1.06	10.93 ± 1.29	9.95 ± 1.24	11.72 ± 1.33	12.95 ± 1.40	2.52	0.11
**Eating**									*χ^2^*	*p*
Strain	CONV	SG								
	11.6 ± 0.62	11.5 ± 0.62							0.01	0.31
Stocking Density	LO	HI								
	11.6 ± 0.62	11.5 ± 0.62							0.01	0.93
Age	25d	39d								
	14.1 ± 0.45^a^	9.0 ± 0.45^b^							**675.57**	**<0.0001**
Strain Stocking Density	CONVLO	CONVHI	SGLO	SGHI						
	11.3 ± 0.87	11.9 ± 0.87	11.9 ± 0.87	11.2 ± 0.87					0.49	0.49
Stocking Density	LO	LO	HI	HI						
Age	25d	39d	25d	39d						
	14.1 ± 0.63	9.1 ± 0.63	14.2 ± 0.63	8.8 ± 0.63					1.43	0.23
Strain Stocking Density	CONVLO	CONVLO	CONVHI	CONVHI	SGLO	SGLO	SGHI	SGHI		
Age	25d	39d	25d	39d	25d	39d	25d	39d		
	15.1 ± 0.89	7.6 ± 0.89	15.6 ± 0.89	8.2 ± 0.89	13.0 ± 0.89	10.7 ± 0.89	12.9 ± 0.89	9.5 ± 0.89	2.50	0.11
**Drinking**									*χ^2^*	*p*
Strain	CONV	SG								
	1.95 ± 0.15^a^	1.04 ± 0.11^b^							**24.96**	**<0.0001**
Stocking Density	LO	HI								
	1.25 ± 0.12^b^	1.69 ± 0.14^a^							**5.82**	**0.019**
Age	25d	39d								
	1.52 ± 0.10	1.41 ± 0.10							1.42	0.23
Strain Stocking Density	CONVLO	CONVHI	SGLO	SGHI						
	1.8 ± 0.20	2.1 ± 0.22	0.8 ± 0.13	1.3 ± 0.17					1.07	0.30
Stocking Density	LO	LO	HI	HI						
Age	25d	39d	25d	39d						
	1.4 ± 0.13	1.2 ± 0.13	1.7 ± 0.15	1.7 ± 0.15					1.33	0.25
Strain Stocking Density	CONVLO	CONVLO	CONVHI	CONVHI	SGLO	SGLO	SGHI	SGHI		
Age	25d	39d	25d	39d	25d	39d	25d	39d		
	2.0 ± 0.24	1.6 ± 0.21	2.3 ± 0.25	1.9 ± 0.23	0.8 ± 0.15	0.8 ± 0.15	1.2 ± 0.18	1.5 ± 0.20	1.06	0.30
**Preening**									*χ^2^*	*p*
Strain	CONV	SG								
	5.0 ± 0.24^b^	5.7 ± 0.24^a^							**9.43**	**0.002**
Stocking Density	LO	HI								
	5.6 ± 0.24^a^	5.1 ± 0.24^b^							**4.17**	**0.041**
Age	25d	39d								
	6.0 ± 0.22^a^	4.8 ± 0.22^b^							**72.52**	**<0.0001**
Strain Stocking Density	CONVLO	CONVHI	SGLO	SGHI						
	5.2 ± 0.30	4.8 ± 0.30	6.0 ± 0.30	5.5 ± 0.30					0.06	0.81
Stocking Density	LO	LO	HI	HI						
Age	25d	39d	25d	39d						
	6.3 ± 0.26	4.9 ± 0.26	5.6 ± 0.26	4.6 ± 0.26					1.23	0.27
Strain Stocking Density	CONVLO	CONVLO	CONVHI	CONVHI	SGLO	SGLO	SGHI	SGHI		
Age	25d	39d	25d	39d	25d	39d	25d	39d		
	6.4 ± 0.33	4.0 ± 0.33	5.7 ± 0.33	3.8 ± 0.33	6.1 ± 0.33	5.9 ± 0.33	5.6 ± 0.33	5.4 ± 0.33	1.10	0.30
**Dustbathing**										
Strain	CONV	SG								
	0.13 ± 0.02	0.17 ± 0.02								
Stocking Density	LO	HI								
	0.18 ± 0.02	0.12 ± 0.01								
Age	25d	39d								
	0.16 ± 0.02	0.14 ± 0.02								
Strain Stocking Density	CONVLO	CONVHI	SGLO	SGHI						
	0.16 ± 0.02	0.10 ± 0.02	0.20 ± 0.03	0.14 ± 0.02						
Stocking Density	LO	LO	HI	HI						
Age	25d	39d	25d	39d						
	0.17 ± 0.02	0.18 ± 0.03	0.14 ± 0.02	0.10 ± 0.02						
Strain Stocking Density	CONVLO	CONVLO	CONVHI	CONVHI	SGLO	SGLO	SGHI	SGHI		
Age	25d	39d	25d	39d	25d	39d	25d	39d		
	0.15 ± 0.04	0.16 ± 0.03	0.13 ± 0.03	0.07 ± 0.02	0.19 ± 0.03	0.20 ± 0.04	0.14 ± 0.03	0.13 ± 0.03		
**Foraging**										
Strain	CONV	SG								
	0.25 ± 0.02	0.54 ± 0.04								
Stocking Density	LO	HI								
	0.41 ± 0.03	0.38 ± 0.03								
Age	25d	39d								
	0.41 ± 0.03	0.38 ± 0.03								
Strain Stocking Density	CONVLO	CONVHI	SGLO	SGHI						
	0.31 ± 0.04	0.19 ± 0.03	0.51 ± 0.05	0.57 ± 0.05						
Stocking Density	LO	LO	HI	HI						
Age	25d	39d	25d	39d						
	0.55 ± 0.05	0.28 ± 0.04	0.68 ± 0.05	0.09 ± 0.02						
Strain Stocking Density	CONVLO	CONVLO	CONVHI	CONVHI	SGLO	SGLO	SGHI	SGHI		
Age	25d	39d	25d	39d	25d	39d	25d	39d		
	0.38 ± 0.05	0.25 ± 0.06	0.35 ± 0.05	0.03 ± 0.02	0.72 ± 0.08	0.31 ± 0.04	1.00 ± 0.10	0.14 ± 0.03

**Table 3:** Estimated means (±SE) comparing the behavior of broilers housed at two stocking densities (LO, HI). Dustbathing and foraging were observed too infrequently for statistical analysis; therefore, raw means and standard errors are reported.

	**Stocking Density**					
	LO	HI		*χ^2^*		*p*
**Sitting**	49.60 ± 2.06	52.60 ± 2.06		1.03		0.31
**Lateral Sitting**	3.18 ± 0.47	3.07 ± 0.46		0.03		0.87
**Walking**	2.24 ± 0.13	1.75 ± 0.30		2.24		0.13
**Standing**	11.00 ± 1.22	10.20 ± 1.18		0.44		0.50
**Eating**	11.10 ± 0.86	10.9 ± 0.86		0.01		0.90
**Drinking**	2.54 ± 0.42	3.41 ± 0.42		2.21		0.14
**Preening**	4.50 ± 0.21	4.15 ± 0.21		1.34		0.25
**Dustbathing**	0.18 ± 0.02	0.12 ± 0.01				
**Foraging**	0.41 ± 0.03	0.38 ± 0.03				

### Sitting

Generally, a greater percentage of broilers were observed sitting at 39d (56.8 %) than 25d (54.8 %; χ^2^ = 33.88, *p* < 0.0001; Table 2). However, the age difference was only observed in CONV broilers (39d (58.3 %) vs. 25d (54.5 %); χ^2^ = 26.73, *p* < 0.0001), and no difference was observed for SG broilers among the different ages (*p* > 0.05, Fig. 1A).

Weight-matched broilers differed in the percentage observed sitting with more 39d CONV (58.30 %) observed sitting than 60d SG (43.90 %; χ^2^ = 24.32, *p* < 0.0001; Fig. 2A). Stocking density had no effect on the percentage of weight-matched broilers observed sitting (χ^2^ = 1.03, *p* = 0.31; Table 3).

### Lateral sitting

A greater percentage of CONV broilers were observed lateral sitting at 25d and 39d (3.8 %, 7.3 %) compared to SG broilers (1.2 %, 1.6 %; χ^2^ = 134.06, *p* < 0.0001; Fig. 1B). Strain and age independently affected the percentage of broilers lateral sitting. A greater percentage of CONV broilers observed lateral sitting (5.4 %) than SG broilers (1.4 %; χ^2^ = 47.83, *p* < 0.0001) and a greater percentage of broilers were observed lateral sitting at 39d (4.1 %) than 25d (2.4 %; χ^2^ = 305.09, *p* < 0.0001; Table 2).

A greater percentage of CONV broilers at 39d (7.44 %) were observed lateral sitting than weight-matched SG broilers at 60d (0.38 %; χ^2^ = 114.22, *p* < 0.0001; Fig. 2B). There was no difference between stocking densities for the percentage of age-matched broilers lateral sitting (χ^2^ = 0.03, *p* = 0.87; Table 3).

### Walking

Overall, more SG broilers (2.57 %) were observed walking than CONV broilers (2.04 %; χ²=64.96, *p* = 0.026), and more broilers were observed walking at 25d (2.60 %) than 39d (2.01 %; χ²=28.88, *p* < 0.0001; Table 2). The percentage of CONV and SG broilers walking differed at 39d (1.60 % vs 2.44 %) but not at 25d (2.50 % vs 3.69 %). Fewer 39d CONV-HI broilers were observed walking (1.26 %) than all other strain, stocking density, and age combinations (χ²=12.72, *p* = 0.0004), but did not differ from 39d CONV-LO broilers stocked at (1.97 %, *p* > 0.05; Table 2). There was no effect of stocking density on the percentage of broilers observed walking (χ² = 0.01, *p* = 0.91; Table 2).

More SG broilers at 60d (2.4 %) were observed walking than CONV broilers at 39d (1.6 %; χ² = 6.03, *p* = 0.01; Fig. 2C). There was no effect of stocking density on the percentage of broilers walking when matched for body weight (χ² = 2.24, *p* = 0.13; Table 3).

### Standing

There was an interaction between stocking density and age (χ^2^ = 8.51, *p* = 0.003), but there were no significant pairwise comparisons when adjusted post-hoc for multiple testing (*p* > 0.05, Table 2). The percentage of LO broilers that were observed standing tended to be higher at 25d (9.18 %) than 39d (8.42 %; *z* = 2.46, *p* = 0.067). When matched for age, a greater percentage of SG broilers (11.37 %) were observed standing than CONV broilers (7.19 %; χ^2^ = 12.69 *p* = 0.0004; Fig. 1D). There were no other main factors or interactions for the percentage of broilers standing (*p* > 0.05, Table 2).

Twice as many 60d SG broilers (14.86 %) were observed standing compared to 39d CONV broilers (7.05 %; χ^2^ = 46.64, *p* < 0.001; Fig. 2D). However, stocking density had no effect on the percentage of weight-matched broilers observed standing (χ^2^ = 0.44, *p* = 0.50, Table 3).

### Eating

A greater percentage of CONV broilers (15.3 %) were observed eating than SG broilers at 25d (12.9 %; χ^2^ = 139.01, *p* < 0.0001), but this difference was not observed at 39d (Fig. 1E). Overall, a greater percentage of broilers were observed eating at 25d (14.1 %) than 39d (9.0 %; χ^2^ = 675.57, *p* < 0.0001).

At the same body weight, almost twice the percentage of 60d SG broilers (14.14 %) were observed eating than 39d CONV broilers (7.85 %; χ^2^ = 30.08, *p* < 0.0001; Fig. 2E). However, stocking density did not affect the percentage of weight matched broilers observed eating (χ^2^ = 0.01, *p* = 0.90; Table 3).

### Drinking

The age-match analysis showed that a greater percentage of CONV broilers (1.95 %) were observed drinking than SG broilers (1.04 %; χ^2^ = 24.96, *p* < 0.0001; Fig. 1F). Additionally, a greater percentage of HI broilers (1.69 %) were observed drinking than LO broilers (1.25 %; χ^2^ = 5.82, *p* = 0.019; Table 2).

When matched for body weight, there were no differences for strain (χ^2^ = 1.04, *p* = 0.31; Fig. 2F) or stocking density (χ^2^ = 2.21, *p* = 0.14; Table 3).

### Preening

No difference was observed in the percentage of broilers preening at 25d; a greater percentage of SG broilers at 39d (5.6 %) were observed preening than CONV broilers at the same age (3.9 %; χ^2^ = 47.78, *p* < 0.0001; Fig. 1G). Overall, more broilers were observed preening at 25d (6.0 %) than 39d (4.8 %; χ^2^ = 72.52, *p* < 0.0001), and at LO (5.6 %) than HI stocking densities (5.1 %; χ^2^ = 4.17, *p* = 0.041; Table 2). Finally, more SG were observed preening (5.7 %) than CONV broilers (5.0 %; χ^2^ = 9.43, *p* = 0.002; Table 2) across both ages.

A greater percentage of 60d SG broilers were observed preening (5.51 %) compared to 39d CONV broilers (3.25 %; χ^2^ = 57.61, *p* < 0.0001; Fig. 2G). Stocking density did not affect the percentage of weight-matched broilers observed preening (χ^2^ = 1.34, *p* = 0.25; Table 3).

### Foraging and dustbathing

Due to the low frequency of dustbathing and foraging behavior observed in this study, the raw means are only described for age-matched broilers in Table 2 and weight-matched, 39d CONV and 60d SG, broilers in Table 3.

For age-matched broilers, the greatest percentage of dustbathing was observed in 39d SG-HI broilers (0.20 %), and the lowest percentage was observed in 39d CONV-HI broilers (0.07 %; Fig. 1H). A similar percentage of weight-matched CONV and SG broilers were observed dustbathing (0.13 % vs 0.17 %; Fig. 2H), while a greater percentage of dustbathing was observed at LO than HI densities (0.18 % vs 0.12 %).

The greatest percentage of foraging was found in 29d SG broilers (Fig. 1I). Foraging was observed least in 39d CONV-HI broilers (0.03 %), and the greatest percentage was observed in 25d CONV-HI broilers (1.00 %; Table 2). A greater percentage of SG broilers (0.54 %) were observed foraging than CONV broilers (0.25 %; Fig. 2I).

## Discussion

The behavior of conventional and slow-growing broilers at the same age and body weight remains underexplored in scientific literature and this gap limits the information available to stakeholders to make informed decisions about broiler welfare standards and production practices. This study highlighted that strain, age, and body weight have differential effects on the behavior of broilers. Many studies have compared conventional and slow-growing broilers, but few have incorporated different stocking densities into the design. Independent of age and stocking density, more slow-growing broilers were observed walking, standing, and preening, while more conventional broilers were observed drinking and lateral sitting. As conventional broilers aged, walking, eating, and preening decreased, and sitting and lateral sitting increased, while there were no consistent behavioral patterns as slow-growing broilers aged. We also compared broilers matched for body weight, 39d conventional and 60d slow-growing, and found that conventional and slow-growing broilers at the same body weight behaved differently for all the behaviors we observed, except drinking behavior. These observations suggest that differences in behaviors between conventional and slow-growing broilers may not be solely driven by differences in body weight, but also by the interplay between age and body weight.

The slow-growing strain of broilers used in our experiment exhibited more active behaviors than the conventional broilers which agrees with other reports (Bokkers and Koene, 2003; Ghayas et al., 2021; Abeyesinghe et al., 2021; de Jong et al., 2021; Dawson et al., 2021). Slow-growing broilers at market weight (60d) were more active than conventional broilers at the same body weight (39d). This suggests that the decrease in activity may not be related to weight, but possibly to the differences in body conformation. A study of the body conformation of the broilers used in our experiment indicated that slow-growing broilers had narrower but longer shanks than conventional broilers (Weimer et al., 2020). Similarly, in a study evaluating 12 conventional and slow-growing broiler strains, the four conventional strains had shorter tibias with narrower diameters than the eight slower-growing strains (Santos et al., 2022). We also previously reported that conventional broilers from this study had shorter keel lengths and greater pelvic and breast widths than slow-growing birds in this study (Weimer et al., 2020). It has been suggested that wider pelvises in broilers can result in a crouched posture, with the center of mass positioned more cranially (Paxton et al., 2013). The increased breast yield in conventional broilers, along with a wider pelvis, also accentuates this shift in the center of mass to a cranial position. These factors may contribute to less active behaviors in broilers, such as walking and standing. Another potential explanation is that the behaviors occur less frequently or for a shorter duration due to the increased energy requirement to maintain leg extension in standing or leg swing in walking (Paxton et al., 2013). Therefore, a combination of differing body conformation, lower breast yield, and market weight age, as demonstrated in related studies (Weimer et al., 2020, 2022), may have contributed to the increased active behaviors (walking and standing) of slower-growing broilers. We found that as birds aged, conventional broilers showed a decrease in active or load-bearing behaviors such as walking and standing. In contrast, inactive behaviors such as sitting and lateral sitting increased.

Unlike conventional broilers, the decrease in active behaviors and increase in inactive behaviors was not observed in slow-growing broilers, thus supporting our hypotheses. Previous studies, such as Dawson et al. (2021), also showed that conventional broilers decreased walking over time, whereas slow-growing broilers walked more than conventional, though they also decreased with age. Our data, however, showed no decrease in walking among slow-growing broilers. Bokkers and Koene (2003) demonstrated that the percentage of conventional broilers walking decreased sharply at three weeks of age, whereas similar declines in walking were not observed in slow-growing broilers until 6 weeks of age. The decrease in walking observed in that study was accompanied by a corresponding increase in sitting in both conventional and slow-growing broilers (Bokkers and Koene, 2003), which further corroborates our data.

The conventional broilers in our study were observed lateral sitting more than the slow-growing broilers. This supports the findings of Abeyesinghe et al. (2021), who also observed that conventional broilers performed more lateral sitting (side-lying) than slow-growing broilers. The increase in lateral sitting was reflected in the higher incidence of hock burn reported previously from this study for the conventional broilers stocked at the lower density (29 kg/m^2^) that were 53.2 % more likely to have hock burn compared to slow-growing broilers stocked at the same density (Weimer et al., 2020), that may be partially explained by slow-growing broilers remaining active throughout the grow-out period. Similarly, Rayner et al. (2020) reported an increased prevalence of hock burn and pododermatitis in conventional versus slower-growing broiler strains, which was associated with decreased activity (play and exploration) in conventional strains. This was also observed by Dixon (2020), who found that the slower-growing strain was more active (standing, walking, foraging) and had lower hock burn scores than the three conventional strains used.

Preening behavior differed among genetic lines, with a greater percentage of slow-growing broilers preening than conventional broilers. These findings are in agreement with those of Abeyesinghe et al. (2021), reporting that slower-growing strains of broilers exhibited more comfort behaviors (preening and dustbathing) at 29d than conventional broilers. The greater occurrence of preening in slower-growing broilers compared to conventional broilers was also observed in Dixon (2020). However, some conflicting research about preening behavior in conventional and slow-growing broilers exists. Bokkers and Koene (2003) found that conventional and slow-growing broilers performed a similar amount of preening up to six weeks of age, whereas from seven to twelve weeks of age conventional broilers performed a greater percentage of preening. Our lack of observed differences due to stocking densities, but rather due to strains, strongly suggests that behavioral differences are primarily related to genetics – possibly genetic factors associated with body conformation, as discussed above.

There were only a few effects of stocking density on broiler behavior. We found that a greater percentage of broilers were observed drinking at the higher stocking density, and more preening occurred at the lower stocking density. However, we must interpret these results with caution, as a 1 % difference in the percentage of broilers observed drinking equates to fewer than one bird per pen, which may lack biological significance. Although, Kaya and Dereli Fidan (2023) also reported more incidences of drinking at a higher stocking density (18 birds/m^2^) than a lower stocking density (12 birds/m^2^). We found that a greater percentage of conventional broilers were observed drinking than slow-growing broilers, which is consistent with previous literature (Bokkers and Koene, 2003; Wallenbeck et al., 2016; van der Eijk et al., 2022). Our eating results did not correspond to drinking behavior. Overall, we found no consistent trend in the percentage of broilers observed eating, at 25d a higher percentage of CONV were observed eating than SG and the inverse was found when comparing broilers at the same market weight. Our findings partially contradict some other literature, which found that a higher percentage of conventional broilers were observed eating than slow-growing broilers (Bokkers and Koene, 2003; Wallenbeck et al., 2016; van der Eijk et al., 2022). This contradiction may be, in part, due to most literature comparing conventional and slow growing broilers at the same age. By comparing broilers strains at the same body weight (market weight) this may highlight a different aspect of feeding behavior and complement studies which show that slow-growing broilers have poorer feed efficiency and therefore need to eat more to reach market weight than conventional broilers particularly during the finisher period (Dixon, 2020).

We found little other effects of stocking broilers at 29 or 37 kg/m^2^ on the percentage of broilers performing various behaviors between slow-growing and conventional birds. This does not support the findings of van der Eijk et al. (2022), who reported that a greater percentage of broilers stocked at 24 kg/m^2^ were observed performing active behaviors, including walking, foraging, and preening than broilers stocked at 42 kg/m^2^. However, this difference may be explained by the high stocking density in van der Eijk et al. (2022) being 5 kg/m^2^ more than the HI stocking density in our study. The occurrence of dustbathing in our study was low. One limitation of this research is the use of scan sampling, which, while minimizing bias by observing the entire population (Daigle and Siegford, 2014), may miss rare or brief behaviors not visible at the group level or outside the sampling interval. Regardless of the low occurrence of dustbathing, a numerically higher percentage of broilers were observed dustbathing at the lower stocking density (29 kg/m^2^), which is in concordance with previous literature (Dixon, 2020; de Jong et al., 2021). Other researchers have demonstrated limited effects of stocking density on the behavior of broilers (Son, 2013; Shynkaruk et al., 2023; Evans et al., 2023). Evans et al. (2023) found that increased environmental complexity, rather than decreasing stocking density, was a better predictor of increased activity in broilers. Another study assessing 20d conventional broilers did not show significant effects of stocking density, ranging from 31 to 41.5 kg/m^2^, on active or exploratory behaviors but did find a linear increase in resting behavior at higher stocking densities (Shynkaruk et al., 2023). Overall, our results indicate that stocking density had minimal impact on the behavior of broilers regardless of whether they were slow-growing or conventional. However, understanding the effects of stocking density on broiler behavior is complicated by factors beyond space availability alone.

One of the challenges when assessing the space use of broilers and ideal stocking densities is that broilers do not equally space themselves from each other. Instead, they tend to cluster within barns, creating areas of high and low densities of broilers within a given pen or barn (Febrer et al., 2006). Clustering is also observed in laying hen pullets (Keeling et al., 2017). Additionally, chickens exhibit behavioral synchrony, which is when a large proportion of the flock engages in the same behavior at the same time, including feeding, dustbathing, and resting (Alvino et al., 2009; Keeling et al., 2017). Thus, birds are not randomly distributed throughout their space and instead cluster in groups throughout the barn (Febrer et al., 2006), which provides a challenge to ensure that all birds have adequate space to access resources simultaneously. Although increasing stocking density may not alter the percentage of broilers resting, higher stocking densities may increase the number of disturbances that broilers experience while resting and, therefore, affect their quality of rest (Febrer et al., 2006).

This experiment highlights behavioral differences between slow-growing and conventional broiler strains, identifying key behavioral differences such as lateral sitting in the current study. Future research should avoid grouping behaviors, such as lateral sitting, into broad categories, such as “sitting” or “inactive,” as this can obscure differences between treatment groups. When studying the differences between slow-growing and conventional broilers, future research should explore multiple genetic strains to investigate the effects of stocking density on broiler behavior. It remains challenging to assess the relationship between growth rates, body confirmation, and leg health (Weimer et al., 2020) and to determine which factors most contribute to the observed differences in activity between slow-growing and conventional broilers.

## Conclusion

We aimed to assess the effects of stocking density on the behavior of one conventional and one slow-growing broiler strain. More conventional broilers were observed sitting and lateral sitting than slow-growing broilers, while more slow-growing broilers were observed standing, walking, and preening compared to conventional broilers. There were minimal effects of stocking density on the percentages of birds performing various behaviors, suggesting that growth rate or body conformation, and not stocking density, was a stronger predictor of behavior in our study. Studies comparing different genetic strains of broilers should assess behavior at multiple ages and compare behavior at similar chronological and biological ages.

## Declaration of competing interest

The authors declare there are no conflicts of interest.
